# New Methodology to Evaluate and Optimize Indoor Ventilation Based on Rapid Response Sensors

**DOI:** 10.3390/s24051657

**Published:** 2024-03-04

**Authors:** María del Mar Durán del Amor, Antonia Baeza Caracena, Francisco Esquembre, Mercedes Llorens Pascual del Riquelme

**Affiliations:** 1 Department of Chemical Engineering, Faculty of Chemistry, Campus de Espinardo, University of Murcia, 30100 Murcia, Spain; mariamar.duran@um.es (M.d.M.D.d.A.); abaeza@um.es (A.B.C.); llorens@um.es (M.L.P.d.R.); 2 Department of Mathematics, Faculty of Mathematics, Campus de Espinardo, University of Murcia, 30100 Murcia, Spain

**Keywords:** rapid response sensors, RRS, ventilation, air changes per hour, ACH, indoor air quality

## Abstract

The recent pandemic increased attention to the need for appropriated ventilation and good air quality as efficient measures to achieve safe and healthy indoor air. This work provides a novel methodology for continuously evaluating ventilation in public areas using modern rapid response sensors (RRS). This methodology innovatively assesses the ventilation of a space by combining a quantitative estimation of the real air exchange in the space—obtained from CO_2_ experimental RRS measurements and the characteristics of and activity in the space—and indoor and outdoor RRS measurements of other pollutants, with healthy recommendations from different organisations. The methodology allows space managers to easily evaluate, in a continuous form, the appropriateness of their ventilation strategy, thanks to modern RRS measurements and direct calculations (implemented here in a web app), even in situations of full activity. The methodology improves on the existing standards, which imply the release of tracer gases and expert intervention, and could also be used to set a control system that measures continuously and adapts the ventilation to changes in indoor occupancy and activity, guaranteeing safe and healthy air in an energy-efficient way. Sample public concurrence spaces with different conditions are used to illustrate the methodology.

## 1. Introduction

Human populations of industrialised countries spend up to 90% of their daily time indoors [1]. Therefore, ensuring good indoor air quality (IAQ) has been an important component of public health policies for years [2]. Poor IAQ has been shown to be associated with a wide range of adverse human health effects and high mortality rates [3,4,5]. Poor indoor air quality can also have a severe economic impact, damaging valuable objects in libraries, archives, or museums [6], as well as reducing employee productivity by 10% to 15% in work environments and reducing students’ academic performance [7,8].

A recent comment article in Nature has highlighted the need for technical guidelines to evaluate the effectiveness of ventilation systems to achieve safe and healthy indoor air. This evaluation should also consider energy efficiency and savings [9].

Maintaining adequate ventilation has been shown to be an essential tool to control indoor air pollution levels [10]. Indoor air quality in buildings is greatly affected by internal factors such as the number of occupants and their activity; furniture or construction materials; heating and ventilation air-conditioning (HVAC) systems; other mechanisms of ventilation; thermal comfort conditions; heating sources; etc. [5,11,12,13]. There are many ways to provide or increase indoor ventilation rates. Ventilation can be natural, taking advantage of the distribution of the space and the locations of the doors and windows in the space, or it can be forced or mechanical by creating an artificial air flow using instrumentation. In the second case, the air can be introduced from the outside (impulsion) or removed from the room (extraction). There is also the option of configuring a centralized forced ventilation system or HVAC in a way that a percentage of outside air is introduced, and the rest is recirculated.

However, during recent decades, the rehabilitation (or remodelling) and new designs of buildings have aimed at effective energy savings. This involves airtight, well-insulated, and sealed constructions that limit natural ventilation or make buildings completely dependent on mechanical ventilation to recirculate indoor air, with a greatly reduced level of outdoor air dilution [11,14,15,16]. On the other hand, the HVAC systems of old buildings are often technologically obsolete, favouring the increase in pollution levels in the interior [17]. All these factors can cause the average indoor concentration levels of certain pollutants to be two (and even five) times higher than at the outside [1,11]. To address this, a recently studied focus is variable air volume systems, such as air terminal devices (ATDs) with changing geometry, which have been demonstrated to reduce contaminant migration, improving indoor air quality [16].

Internationally recognized institutions have issued guidelines with intervals of recommended numbers of air changes per hour (ACH) for different types of installations and uses, which will typically guarantee good indoor air quality as well as protection against viruses in general [18]. These recommended intervals are aimed at the design of ventilation systems (which explains why the intervals are, in many cases, rather wide), and national bodies use these guidelines when issuing regulations for new constructions. These guidelines are summarised in a much-cited list of recommended ACH intervals for different indoor spaces, depending on their use [19]. Table 1 displays a selection of the recommendations for some spaces of interest for this paper.

However, having installed a correctly dimensioned ventilation system does not guarantee its effective nor efficient everyday use. The recommendations alone, bluntly taken, do not consider factors such as the air volume of the space or its actual occupancy. Also, in mild weather conditions, it seems reasonable to help ventilate by just opening windows. The effectiveness of this approach depends on the geometry of the windows, their orientation with respect to the external wind direction, and whether they are in opposite sides of the building, creating air currents.

The advent of the COVID-19 pandemic brought increased public attention to the importance of the actual ventilation rate of indoor locations. A great deal of respiratory diseases are transmitted mainly by air, through aerosols [20,21,22]. This means that the risk of transmission of airborne diseases increases noticeably in (poorly ventilated) indoor and semi-indoor spaces. A series of anti-COVID-19 recommendations, including increasing the ventilation rates, are in some case more restrictive than those prescribed by the existing IAQ directives. More details about this are given in Section 2.

But, to complicate things, there is also ample scientific evidence that establishes a positive contribution of environmental pollution to the spread and lethality of COVID-19 and other viral infectious diseases [23]. In particular, the incidence of the virus is closely associated with the exposure to PM_2.5_ and NO_2_ [1,24]. It has been found that poor air quality favours transmission and accelerates the spread of the virus, worsens health outcomes in COVID-19 patients, and increases the mortality rate in people with respiratory diseases, people with cancer, or those older than 60 years [1,24,25,26].

In summary, improving ventilation in closed spaces is not only typically the simplest and most economical measure to improve IAQ [1,11,27], but can also be a key element to reduce exposure to aerosols in confined areas, limiting the spread of the SARS-CoV-2 virus and other infectious agents [12,28]. However, a high ventilation rate will not always be guaranteed to eliminate the viral load, especially in scenarios where there are constantly changing external circumstances and dynamic internal activities and occupants. And, even worse, ventilation itself can also be a source of contamination and exposure when outdoor pollutant concentrations are high, such as in areas with heavy traffic or near industrial activities [11], not to mention the increase in energy consumption involved.

The conclusion is that establishing a ventilation strategy that ensures a sufficiently high rate of air exchange and, in turn, allows for the maintenance of thermal comfort conditions and indoor pollution levels at optimal concentrations for human health is a complex task, beyond the original design of the ventilation system. There is, therefore, a need for updated methodologies and ventilation strategies that address the post-COVID-19 requirements, adequately combining the recommended air changes per hour (ACH) with the control of indoor air quality levels in the most energy-efficient way. Such a methodology should continuously assess the actual ventilation taking place in each space at a given time, considering the characteristics of the space, its occupancy, the activity inside it, the ventilation strategy, and the outside concentration of pollutants (from which the renovation air is being taken).

This paper addresses this problem by providing a novel methodology to continuously evaluate the efficiency of the existing ventilation strategy and the quality of indoor air in different scenarios using modern rapid response sensors.

To date, ventilation evaluation methodologies are carried out, in most cases, according to the procedure defined in the ASTM E741-11 Standard Test Method for Determining Air Change in a Single Zone by Means of a Tracer Gas Dilution. This test method covers techniques (concentration decay, constant injection, and constant concentration) using tracer gas dilution for determining a single zone’s air change with the outdoors, as induced by weather conditions and by mechanical ventilation [29]. Using this test requires knowledge of the principles of gas analysis and instrumentation and the correct use of a series of complex formulas, so it cannot be carried out by non-specialists. However, the main limitation of this standard is that the results from this test method pertain only to those conditions of weather and zonal operation that prevailed during the measurement and cannot be easily extrapolated to different conditions. This implies the need to carry out a new test every time the characteristics or conditions of the space studied are modified, which further complicates the task of evaluating ventilation and does not allow for short-term corrections.

In this context, the use of modern rapid response sensors (RRS) represents a practical benefit compared to traditional evaluation methods. RRS devices are becoming very popular because they offer numerous advantages, such as cost, speed of response, easy and comfortable use and application, ergonomics, versatility, etc. [30,31,32].

Section 2 describes in detail the methodology proposed in this paper. The methodology includes a quantitative method to easily estimate the actual air changes per hour in a space at a given moment from its characteristics, occupation, and the activity inside it, using RRS measurements of the concentrations of CO_2_ in the inside and outside air. Although the calculation is based on the well-known box mass balance model, it is used here in an innovative way by relating it to the international ACH recommendations in a dynamical form. A web app has been developed and implemented in this work to help perform the calculations involved. The methodology also studies air quality by comparing the RRS-measured experimental concentrations of atmospheric pollutants inside and outside the space, checking whether the recommended exposure limit values established by the different regulations are respected. The combined consideration of both studies can then lead to proposals for improvement and control actions (both in ventilation and energy consumption), or—in very particular situations—to signal important incompatibilities that may require infrastructural changes. Another important question, also covered in Section 2, is that of compiling the various recommendations, some new, that have been issued by different organisms concerning the maximum concentrations of different pollutants or the recommended respiration flow rate per person (the amount of air that should be made available to each person) to ensure good air quality and protection against some particularly dangerous viruses, such as SARS-CoV-2 coronavirus.

To illustrate the use and validity of the proposed methodology to help to achieve this combination, a number of case studies are considered in Section 3: two study rooms, a coffee shop, a restaurant, a cinema room, a sports gym, and an office in a car repair establishment were taken as examples with very different characteristics and uses. Ventilation conditions (depending on CO_2_ concentrations) and air quality (depending on PM_2.5_, PM_10_, VOCs, and NO_2_ levels) were studied in these public concurrence spaces after the start of the new normality due to the COVID-19 health crisis in Spain, in the city of Murcia, located in the southeast of the country. The measurements obtained using rapid response sensors are considered in combination, using the proposed methodology, to assess the ventilation strategy and indoor air quality.

The results obtained in this study help to establish a basis to suggest ventilation improvements for most spaces of public concurrence, to prevent the risk of spread and contagion by SARS-CoV-2 while minimizing exposure of their occupants to atmospheric contaminants, as well as optimizing energy expenses. The continuous evaluation methodology facilitated by modern rapid response sensors, and the immediate improvement actions derived from it, provide guidelines that can be applied to other spaces or to future scenarios should a similar pandemic occur again. This methodology, therefore, is a novel tool with easy applicability that allows even non-specialists to easily and continuously assess the ventilation system of a space. Managers can use this continuous computation to adapt the general ventilation to changes in indoor occupancy and activity, thus guaranteeing the provision of safe and healthy air in an energy-efficient way at all times. These and other conclusions are given in Section 4.

## 2. Materials and Methods

### 2.1. Studied Spaces

For the evaluation of ventilation efficiency and air quality, seven different public concurrence spaces, representative of work, student, and social or leisure environments, were selected. These types of spaces were all related to numerous outbreaks of COVID-19 contagion [33]. As a work environment, an office inside a car repair installation was chosen. As student locations, two study rooms were studied, one at a city library and another at the University of Murcia. Finally, a coffee shop, a restaurant, a cinema, and a sport gym were selected as leisure spaces. All these spaces were located downtown in the city of Murcia (Spain).

### 2.2. Sensors

To measure air pollution levels, the portable air quality sensor meter Flow 2.0, v2018 version, designed by Plume Labs in partnership with the Frog studio, was used [34]. Flow 2.0 can measure, every minute, the air quality indices (AQI) and the air concentration levels of different pollutants: nitrogen dioxide (NO_2_) and volatile organic compounds (VOCs) in ppb *v*/*v*, as well as particulate matter equal to or less than 10 and 2.5 microns (PM_10_ and PM_2.5_, respectively) in ${\mu g\;m}^{-3}$. Although the manufacturer guarantees the precision and reproducibility of the sensor measurements [35], the portable gauges were validated in the laboratory in a dynamic reference chamber which generated controlled atmospheres [36,37,38,39]. The measurements provided by the Flow 2.0 were controlled by measurements taken with the continuous analysers Thermo model 42i for NO–NO_2_–NO_x_, 48i for CO, 49i for O_3_, and 450i for SO_2_–H_2_S, and Syntech Spectras GC955 gas chromatograph for other chemicals. The validation results and measurement errors of the sensors can be accessed at https://doi.org/10.6084/m9.figshare.25272541.

Flow 2.0 needs to be linked to a mobile phone via a software application. This allows for real-time monitoring of the quality indices of all pollutants on the device’s screen while being recorded on the platform, along with the measured air concentration levels. After finishing the sampling, all of the data can be exported in Excel format to an email account linked to the device for processing. This Excel file contains the measured AQI and concentrations of the pollutants with their corresponding date, time, and geographical location.

The multifunctional VISLONE Air Quality Monitor was used to determine the ambient CO_2_ concentration in ppm [40]. Each measurement lasted ten minutes. During this time, the meter continuously recorded the CO_2_ concentration and finally provided the average value on its digital display.

An optical distance meter was used to obtain the dimensions of the studied spaces whose dimensions were unknown. The integrated laser TLM 165 model designed by STANLEY^®^ [41] was chosen for these measurements.

### 2.3. Methodology

#### 2.3.1. Sampling Strategy of Pollutants Measurement

**Figure 1:**
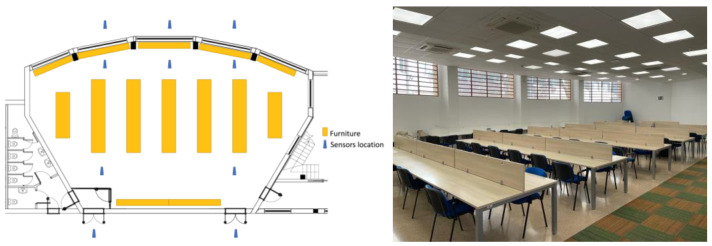
Map with sampling points and photograph of the university library.

The sampling of atmospheric pollutants (PM_2.5_, PM_10_, VOCs, and NO_2_) and CO_2_ in the studied spaces was carried out simultaneously with their respective devices, following a series of criteria and considerations:

- Aerosol sprays such as deodorants or perfumes, disinfectant gels, and any other type of personal care products could not be used before handling the Flow 2.0 device. These substances cause important alterations in the measured values of pollutants, mainly VOCs, since they are important emission sources of them [11].
- In every space, there was enough ventilation to guarantee an even mixture of air. This was experimentally verified by measuring at different points of each space (see Figure 1 below). Measurements were always taken once the sensors stabilized to achieve a steady state.
- The devices were placed as far away from doors and windows as possible, in a central position in the space, at a minimum distance of 1 metre from people and at a height of 1 to 1.5 m from the floor. This placement was important in order to avoid external disturbance factors during sampling, such as emitting sources (i.e., human), the airflow from the HVAC system, or make-up air from windows, and to prevent misestimating the actual concentration [42].
- The number of people present in each place during the measurements was recorded.
- Measurements were taken both inside and outside the different studied spaces.
- When the measuring devices were switched on, it was necessary to wait for at least 10 min to ensure that the levels of pollutants monitored by the meters stabilized. After that, the concentration values were recorded for another 10 min. With the data obtained, the mean was calculated and taken as the pollutant concentration value in a stationary state for the calculations.

The sampling campaign was carried out during the month of October 2021. During those days, outside temperatures were pleasant, resulting in natural high thermal comfort inside the studied spaces. Therefore, the heating or air conditioning systems were disconnected inside the spaces during measurements. On the other hand, there were no precipitations or gusts of wind that could alter the measurements outside, and the levels of humidity and radiation were optimal for sampling. To illustrate the sampling locations, Figure 1 shows the case of the university library.

Finally, when obtaining the dimensions of the studied spaces, the meter was placed as close as possible to the walls and floor, and special care was taken to ensure that there were no objects in the way that could diffract and/or deflect the laser.

#### 2.3.2. Ventilation Assessment

Ventilation in each studied space was evaluated according to the CO_2_ concentration measured inside the premises, and this was used to estimate the actual air changes per hour (ACH) taking place. Unlike other pollutants, CO_2_ is produced almost entirely by human breathing and is highly stable in the environment. This makes CO_2_ a commonly used indicator of the occupation and emission of aerosols by people and of the rates of air exchange in indoor spaces [42,43,44,45]. In our study, high levels of CO_2_ are associated with low ventilation rates and, therefore, with potential risk of SARS-CoV-2 infection [46,47,48].

##### Estimation of the Concentration of CO_2_ in the Stationary State and the Real ACH

In order to assess the efficiency of ventilation in each space and situation, we first derive a quantitative formula based on the dilution principle that interrelates the expected CO_2_ concentration in stationary state, $C_{SS}\;,$ inside a given ventilated space with its flow rate of external air, $F_{ext}$; the external air CO_2_ concentration, $C_{o}$; and the space’s internal CO_2_ average generation rate, $G_{CO_{2}}$.

One of the principles of ventilation is the dilution effect principle. During this dilution, a mass flow between polluted and clean air is produced, and the “black box model” is used to explain the balance of matter in the space under study [49,50]. In this model, the mass balance of the interest component, CO_2_ in this case, in the space is considered:

Outdoor air entrance + Generation of CO_2_ inside = Accumulation of CO_2_ inside + Exit of air

Assuming that the chemical agent is perfectly diluted in the ambient air, this mass balance can be expressed in the form of the following differential equation:

(1) $$V\cdot \frac{dC_{i}}{dt}=Q_{e}\cdot C_{e}+10^{6}G-Q_{s}\cdot C_{s}$$

**Figure 2:**
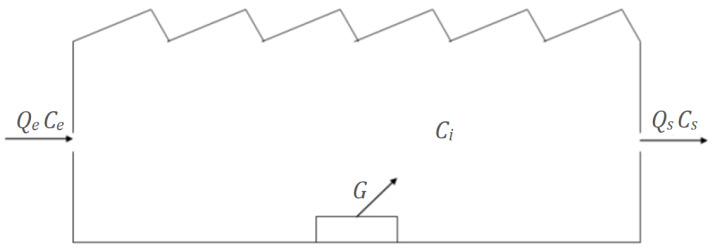
General dilution ventilation in a place where a chemical agent is released.

In this equation, V is the total air volume of the space (measured in $m^{3}$); t is time (in hours, h); Q is the volumetric flow of air at the entrance, $Q_{e}$, and exit, $Q_{s}$, of the space (in $m^{3}{\;h}^{-1}$); and C is the concentration (in ppm (*v*/*v*)) of the pollutant at the entrance, $C_{e},\;$ exit, $C_{s},$ and inside, $C_{i}\;$, of the studied space. Finally, G is the CO_2_ generation rate inside the space (in $m^{3}\;h^{-1}$). See Figure 2.

In principle, all magnitudes in this differential equation can have different values and may change in time. However, when a chemical agent is released into the environment of a space and is to be eliminated by renovation or extraction of air from it, additional considerations apply:

(i) To extract a certain flow of air from the space, it is necessary to introduce the same amount of air into it, ensuring a continuous mass flow. Therefore, the inlet and outlet air flow in the space will be equal: $Q_{e}=Q_{s}=Q$.
(ii) The chemical agent is assumed to be perfectly diluted in the ambient air. Hence, the exhaust air will have the same composition as the air contained in the room: $C_{i}=C_{s}=C$.
(iii) The air volume V of the space is constant, and its inlet air flow Q is typically caused by a constant flow rate, $F_{ext}$, of air coming directly from the outside. This in turn implies that $C_{e}=C_{o}$, where $C_{o}$ is the measured CO_2_ concentration on the outside, which is also assumed to remain constant during the process.

With these assumptions, the differential Equation (1) produces the following initial value problem:

(2) dCtdt=QV·(Co−Ct)+106GtV Ctinit=Cinit

where Q, V, and $C_{o}$ are constants, and $C_{init}$ is the CO_2_ concentration at a given initial time $t_{init}$.

For a reasonable (for instance, continuous) function G, not necessarily constant, the problem has the following unique closed-form solution:

(3) $$C\left(t\right)=C_{o}-\left(C_{o}-C_{init}\right)\;e^{-\frac{Q}{V}\;\left(t-t_{init}\right)}+\frac{10^{6}}{V}e^{-\frac{Q}{V}\;\left(t-t_{init}\right)}\int_{t_{init}}^{t}G\left(s\right)e^{\;\frac{Q}{V}\;\left(s-t_{init}\right)}ds.$$

Complex as it may seem, the first term of the solution is constant and the second term clearly tends to zero as the time increases (quickly, if the ratio $\frac{Q}{V}$ is large). This means that, in the long term, every solution:

(a) in the absence of internal CO_2_ generation (that is, if $G\left(t\right)=0$, for all $t\geq t_{init}$), tends exponentially to the external CO_2_ concentration, $C_{o}$ (as one would expect);
(b) if $G\left(t\right)$ is non-zero, has an extra term which adds to the solution of case a).

With no further information about G, it is not possible to say much more. Notice that the extra term of (b) may fluctuate if $G\left(t\right)$ does.

However, if, for example, G is bounded, $G_{min}\leq G\left(t\right)\leq G_{max}$, for $G_{min}$ and $G_{max}$ suitable constants, it is not difficult to show that:

(4) $$C_{o}+10^{6}\frac{G_{min}}{Q}\leq \underset{t\to +\infty }{\lim}C\left(t\right)\leq C_{o}+10^{6}\frac{G_{max}}{Q}.$$

Hence, for any small, positive number ϵ, if we take a value $C\left(t_{L}\right)$ for $t_{L}$ that is large enough (after the transients of the solutions have died out), we have:

(5) $$10^{6}\frac{G_{min}}{Q}-\epsilon \leq C\left(t_{L}\right)-C_{o}\leq 10^{6}\frac{G_{max}}{Q}+\epsilon .$$

A particular case is that in which G is constant: $G\left(t\right)=G$, for all $t\geq t_{init}$ (which implies $G_{min}=G_{max}=G)$. In this case, the solution with initial condition $C_{ss}=C_{o}+10^{6}\frac{G}{Q}$ is a stable stationary state. That is, the constant function $C\left(t\right)=C_{o}+10^{6}\frac{G}{Q}\;$ is one solution to the differential equation, and all other solutions (for different initial conditions) tend to this constant one as t grows. This fact suggests the following practical procedure.

For the studies considered in this work, an average internal CO_2_ generation rate, $G_{CO_{2}}$, is estimated and considered constant during the measurements [51,52]. In this work, it is also assumed that only exhaled metabolic CO_2_ is generated and no other sources or sinks of CO_2_ are present.

Since Q is due to a ventilation characterized by $F_{ext}$, the desired formula is finally obtained:

(6) $$C_{ss}=C_{o}+10^{6}\frac{G_{CO_{2}}}{F_{ext}}.$$

This formula can be used to estimate the expected stationary-state CO_2_ concentration in a space with known ventilation under the indicated assumptions. But it can also be used to set the required external air input flow needed to keep the long-term CO_2_ concentration below prescribed limits.

It is finally possible to use (6) to interrelate the concentration magnitudes with the number of air changes per hour, ACH, in the space that are caused by the ventilation, using the relation $F_{ext}=ACH\cdot V$. This gives:

(7) $$ACH=10^{6}\frac{G_{{\mathrm{CO}}_{2}}}{V\cdot \left(C_{ss}-C_{o}\right)}.$$

When using this formula to estimate the real air changes per hour, $\;ACH_{real}$, in an experimental setting, $C_{ss}$ can be approximated by measuring the CO_2_ concentration at a reasonable large value of time when the concentration value has considerably stabilized.

##### Estimation of Average Internal CO_2_ Generation Rates

The estimation of the average CO_2_ generation rate due to human activity in the studied spaces needed by Equations (6) and (7) is given by the following formula:

(8) $$G_{CO_{2}}=n\cdot Exh_{rate}\cdot 3600\frac{s}{h}\cdot 0.001\frac{m^{3}}{l}$$

Here, $G_{CO_{2}}$ is the estimated generation rate (measured in $m^{3}h^{-1}$), n is the number of occupants present in each space at the time of the measurements, and $Exh_{rate}$ is the average CO_2_ exhalation rate per occupant (in ${l\;s}^{-1}$).

The CO_2_ exhalation rate depends on the age, gender, weight, and metabolic activity of each person. To determine the CO_2_ generation in each case, the average value of the CO_2_ exhalation rate is used as a function of metabolic activity, considering the average profile of the people present at the time of data collection. The average is calculated for both men and women, unifying them into a single age group between 11 and 49 years old, and subsequently, the average between both genders is estimated. Three different average values are obtained according to whether people are at rest, walking, or performing high-intensity physical activity (Table 2). These values were estimated for a standard European and American reference population. A correction factor should be applied for the distributions of ethnic groups in other populations [53].

##### Guidelines for Ideal Air Changes per Hour

Guidelines for air quality control and prevention of COVID contagion are typically implemented by guaranteeing a minimum objective number of air changes per hour, denoted here by $ACH_{obj}$ in the ventilated space. This value is estimated using the following formula:

(9) $$ACH_{obj}=\frac{F_{b}*n}{V}*3600\frac{s}{h}*0.001\frac{m^{3}}{l}.$$

In this expression, $F_{b}$ stands for the respiration flow rate value per person (in ${l\;s}^{-1}$), which is specified by the guidelines, and depends on the type of space studied, the activity carried out by the people in it, and the ventilation goal (Table 3). The value obtained for $ACH_{obj}$ is then typically rounded up to the next integer, which will be denoted, for clarity, in what follows by $\left[ACH_{obj}\right]$.

Using (9), two objective ACH values can be computed for each space: $ACH_{mv}$ when the goal is an adequate minimum ventilation, and $ACH_{ac}$ for the goal of avoiding COVID contagion. Notice that values in Table 3 imply that $ACH_{ac}$ is always greater than $ACH_{mv}$.

In addition to the guidelines in Table 3, other guidelines recommend a limit of 700 ppm for the value of the CO_2_ concentration to avoid COVID contagion in a closed space [54]. If only air quality is concerned, the WHO sets this limit to 1000 ppm [55]. Therefore, when experimental values above 700 ppm are measured, ventilation will still not be adequate, and it will be essential to further ventilate the room to help prevent COVID contagion, even if $\left[ACH_{ac}\right]$ has been exceeded. In these situations, Equation (7) can be used, replacing $C_{ss}$ with 700, to compute the (greater) required ACH. In cases in which only air quality is concerned, a similar computation is needed, but the maximum allowed concentration of CO_2_ is the recommended 1000 ppm limit value.

**Table 3:** Recommended respiration flow rate per person.

Ventilation	Indoor Space	F_b_ Value (l s^−1^)
Prevent COVID-19 contagion	High aerosol generation by singing, loud speaking, aerobic exercise, or close social distance (cafes, restaurants, gyms, transports)	15 [55]
	Libraries, study rooms, and offices	14 [43,56]
	Other ordinary workplaces or public spaces	10 [55]
Minimum required (low-quality air)	Any space	5 [56]

##### Assessment Procedure

To assess the ventilation of a given space, the procedure is then as follows:

(a) Measure the studied space dimensions—width, length, and height—and calculate the air volume V of the space, in $m^{3}$;
(b) Use the information of occupancy, n, and activity in the space, together with Table 2 and (8), to estimate the average CO_2_ generation rate due to human activity, $G_{CO_{2}}$;
(c) Measure the CO_2_ concentration (in ppm) outside$,\;C_{o}$, and inside$,\;C_{ss}$, of each space for 10 min, once the levels remain relatively constant (stationary state);
(d) Use (7) to estimate the actual ACH, denoted here as $ACH_{real}$, taking place in the space;
(e) Use the information of occupancy and activity in the space, Table 3, and (9) to set the target for air renewals to achieve each of the goals: $\left[ACH_{mv}\right]$ and $\left[ACH_{ac}\right]$;
(f) The results of the last two computations are then compared, and the 700/1000 ppm limit is checked, to assess the ventilation of the space. If $ACH_{real}\geq \;\left[ACH_{ac}\right]$ (which is, in turn, greater than $\left[ACH_{mv}\right]$) and the measured $C_{ss}\leq 700$, the ventilation of the space is considered adequate to avoid contagion and to maintain good air quality. If only maintaining air quality is considered, the criterion for adequate ventilation is then $ACH_{real}\geq \;\left[ACH_{mv}\right]$ and $C_{ss}\leq 1000$. Any other situation would require the implementation of additional measures or the eviction of the space.

These calculations were implemented in a freely accessible web application, accessible at http://www.um.es/airquality/ach_calculator (accessed on 30 January 2024). The application allows the experimental data to be introduced and performs all computations described in this subsection.

#### 2.3.3. Air Quality Assessment

In addition to ventilation based on CO_2_ concentrations, indoor air quality was also evaluated in the surveyed spaces in terms of the main atmospheric pollutants. To determine the quality of the ambient air, the concentration levels of the main pollutants, PM_2.5_, PM_10_, VOCs, and NO_2_, were obtained experimentally, inside and outside the studied spaces, and analysed.

The concentration levels of the pollutant substances inside, $C_{i}$, and outside, $C_{o}$, the spaces were first checked to comply with the exposure limit values established in the regulations (see Table 4). Then, their ratio was computed:

(10) $$I/O=\frac{C_{i}}{C_{o}}$$

A value larger than 1 for this ratio indicates the existence of internal sources of pollutants. These need to be identified, characterized, and considered (together with external ones) before issuing final recommendations for the space. Whenever the maximum permitted contamination levels are exceeded, action and improvement measures must be implemented to control the critical emission points and reduce the concentration to values that do not pose a risk to human health.

**Figure 3:**
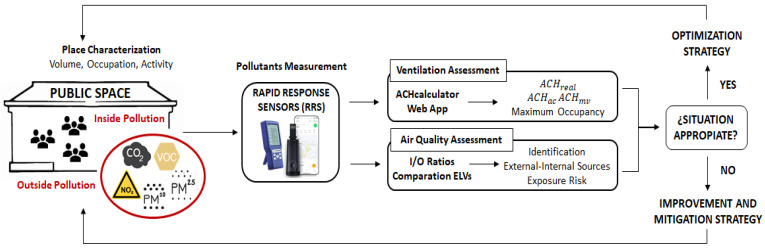
Graphical algorithm of the proposed indoor ventilation assessment procedure.

Figure 3 provides a graphical illustration of the complete assessment procedure.

## 3. Results and Discussion

### 3.1. Ventilation Assessment

The results obtained after applying the methodology for ventilation assessment to the case study spaces are shown in Table 5. The table first lists, in the experimental data section, the measurements obtained for each of the spaces, the estimated $G_{CO_{2}}$ for the occupancy they had during the measurements, and the resulting $ACH_{real}$. For comparison, the next two sections of the table compute the recommended $\left[ACH_{ac}\right]$ and $\left[ACH_{mv}\right]$ for that same occupancy. The type of ventilation is indicated in the last column of the table.

The CO_2_ concentrations measured experimentally, both indoors and outdoors, show great diversity among the studied spaces. Typology and characteristics (occupancy, dimensions, location, ventilation, activity, etc.) of the spaces differ greatly from each other, so it is not possible to identify a pattern or to find a simple association between any of these characteristics and the recorded CO_2_ levels. The cinema room, city study room, and restaurant were the spaces with the highest CO_2_ indoor values, all above 600 ppm. CO_2_ values between 529 and 558 ppm were measured in the coffee shop, office, and university study room. Finally, the gym recorded the lowest levels, with a value around 500 ppm. On the other hand, the concentration of CO_2_ outside the spaces was less than 500 ppm, except in the cases of the office and the coffee shop (a value close to 400 ppm indicates an environment completely free of respiratory aerosols [54]).

As a first global conclusion, none of the spaces exceeded 700 ppm (nor 1000 ppm) for the CO_2_ concentration (the cinema room was very close, though), and all of them, except the cinema room, showed $ACH_{real}\;\mathrm{values}$ larger than the recommended $\left[ACH_{ac}\right]$. The cinema room did not even reach the recommended $\left[ACH_{mv}\right]$.

It is, however, important to note that, during these measurements, the actual occupancy of the spaces was very low. The reason might be that the occupancy was limited due to some, still valid, anti-COVID social distance recommendations. In some cases, the space just happened to have even lower occupancy during the measurements. Therefore, although the computation of the real ventilation still holds, the conclusion that the ventilation is correct cannot be drawn from data in Table 5 alone.

To this end, Table 6 lists the maximum occupancy that each space can support with the estimated $ACH_{real}$ while still observing the recommended limits. For the anti-COVID recommendations, the first section of the table uses (9) to compute the maximum number of people, n; the corresponding internal CO_2_ generation, $G_{CO_{2}}$; and the expected CO_2_ concentration, $C_{ss}$, that will still keep $ACH_{real}\geq \left[ACH_{ac}\right]$ for each space. The section also uses (7) and (8) to compute the maximum n (and expected $G_{CO_{2}}$ and $C_{ss}$) that each space can support with the measured $ACH_{real}$, while keeping the CO_2_ concentration below the limit value of 700 ppm. Similarly, the second section repeats the computations for the recommendations for minimum ventilation.

Table 6 shows that, for anti-COVID recommendations, the cinema room cannot comply with these recommendations for any occupancy. The restaurant is the only other space for which the $ACH_{real}\geq \left[ACH_{ac}\right]$ condition is stricter than the 700 ppm limit. For all others, the maximum occupancy (in bold font) is given by this limit. For minimum ventilation recommendations, except for the cinema room, which, again, cannot comply for any occupancy, the 1000 ppm limit is always the stricter measure and therefore gives the maximum occupancy to choose.

For comparison, Table 7 and Table 8 repeat these computations for each space and guideline using the minimum and maximum recommended ACH for the design of the ventilation systems given in Table 1. A combined inspection of the entries of Table 6, Table 7 and Table 8, together with the expected occupancy for each space, can allow its managers to decide whether the actual ventilation or the installed one is appropriated for the characteristics of each space (in particular, air volume, outside CO_2_ concentration, and activity inside).

### 3.2. Air Quality Assessment

**Figure 4:**
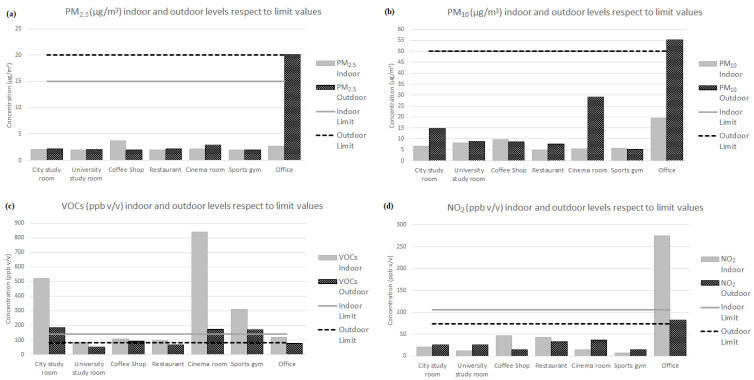
Pollutants’ average measurements compared with regulated limits (Table 4). Indoor and outdoor levels of (**a**) PM_2.5_ (µg m^−3^), (**b**) PM_10_ (µg m^−3^), (**c**) VOCs (ppb *v*/*v*), and (**d**) NO_2_ (ppb *v*/*v*) in each space.

Table 9 shows the experimentally measured mean values of the concentrations, inside and outside, of PM_2.5_ (µg m^−3^), PM_10_ (µg m^−3^), VOCs (ppb *v*/*v*), and NO_2_ (ppb *v*/*v*); their I/O ratio; a description of the location; and possible emission sources of each space. Figure 4 compares the measured average concentration levels of each pollutant with the environmental limit values in Table 4.

Regarding particulate matter, the mean values of PM_2.5_ and PM_10_ in outdoor air were reasonable and within the limits in all spaces except the car office, which showed abnormally high values of 20.17 µg m^−3^ and 55.16 µg m^−3^, respectively. The cinema and the city study room also showed noticeably high outdoor mean PM_10_ values. On the other hand, the internal values were all within limits, with a maximum, slightly high value of PM_2.5_ in the coffee shop of 3.65 µg m^−3^, and a noticeably high value for PM_10_ of 19.46 µg m^−3^, again in the car office. The car office outdoor environment was a car repair facility, which helps to explain these high values, despite an installed air purifier. For all other spaces, urban traffic constitutes the main source of external emissions of particulate matter, which enter the indoor environment through ventilation.

On the other hand, combustion-based appliances, potential internal sources of PM, were identified only in the restaurant and the coffee shop, such as ovens and grills located in the kitchen area. However, in the restaurant, a localized extraction system installed in the kitchen acted directly on these sources, preventing the accumulation of these pollutants. In the remaining spaces, the I/O ratios were less than or very close to 1 (Table 9), which points to the absence of internal sources. Also, the low levels of PM which were measured are a consequence of the low occupancy rates, and the slight differences that could be seen with respect to the outside can be associated with the resuspension of the particles in the air due to the movement of dust [59,60]. With these data, the spaces analysed confirm an adequate level of particulate matter inside and outside, except for in the office.

For VOCs, Table 9 shows that the measured interior levels were higher than the exterior levels in all spaces. Inside, VOCs are emitted by common construction elements and materials such as furniture, carpets and rugs, varnishes, resins, paints, glues, solvents, and different plastics. In addition, cooking and heating devices, cosmetics, and personal care products such as perfumes or deodorants associated with the presence of people, and, in particular, the massive use of cleaning and disinfection products against the SARS-CoV-2 virus, constitute important sources of internal emissions in the spaces studied [11,61,62,63,64]. Outside, the sources of VOC emissions are industrial and combustion processes, as well as, mainly, the burning of fossil fuels during transport.

Comparing the experimental levels obtained with the internal and external limits recommended for this contaminant, which are given in Table 4, the situation is worrisome in some spaces (Figure 4c). The city study room, the cinema room, and the gym exceeded the maximum recommended exposure value both inside and outside of the premises. The cinema produced the maximum value of measured indoor VOCs, 839.55 (ppb *v*/*v*), and I/O ratio, 4.82, which suggests the presence of important interior sources. In addition, as seen in the previous subsection, this space had the worst ventilation (Table 5), which probably favours, in turn, the accumulation of the pollutant in the indoor air, explaining the enormous difference in indoor concentration with respect to the other spaces. The (high) levels measured outside these four places, on the other hand, were very similar, with values from 170 to 184.75 (ppb *v*/*v*), most likely caused by their proximity to heavy traffic, which also contributed to high internal measurements [27,65].

In the other four spaces, the concentrations of VOCs were lower than the internal and external limit standards, except minimally in the case of the coffee shop.

In the case of NO_2_ concentration, all spaces showed indoor and outdoor values within limits, except, once more, the car office, which had abnormally high values of 274.5 and 82.75 (ppb *v*/*v*), respectively, with a surprisingly high I/O ratio of 3.32, which will be discussed in Section 3.3. The coffee shop (3.26) and the restaurant (1.31) also showed I/O ratios higher than 1, which suggests the presence of internal sources associated with cooking activities [66]. In the rest of the spaces, the indoor NO_2_ levels were lower than the outdoor ones, which supports the assumption of an absence of internal sources. Finally, traffic and industrial activities were established as the main sources of external NO_2_ emissions [1,11,66], but they did not represent a risk factor for NO_2_ in these spaces (office excluded).

The results show that outdoor air pollutant concentrations present different values that depend mainly on the location of the studied space. Similarly, indoor pollutant levels are greatly affected by the occupancy rate, activities, and metabolisms of occupants; the characteristics, materials, and constructive elements of the space; the presence of internal and/or external foci; and the effects of ventilation actions. There are great differences in the I/O ratios of pollutants depending on the type of building or space, the local conditions, people’s activities, and even the climate or season of the year. Thus, similarly to what was said in the ventilation assessment subsection, it is not possible to find a direct behaviour pattern or a simple correlation between the levels of pollutants and any one of the factors studied in particular, nor to predict how the contamination will evolve when any of the variables analysed is modified.

### 3.3. Improvement Proposals

After evaluating the ventilation conditions and air quality of all spaces in the present study, each space can then be considered individually to make a more precise diagnosis of the efficiency of its ventilation system, as well as of the possible causes for high values of pollutants, always considering the worst possible scenario (such as the advent of future pandemics). These considerations also allow for the identification of improvement suggestions both in the control or mitigation of sources and in the ventilation system itself. In addition, future scenarios in which the levels of CO_2_, PM, VOCs, or NO_2_ may pose a health risk can also be considered, proposing possible preventive actions.

Any measure that involves reducing pollution levels—the concentration of PM_2.5_, PM_10_, VOCs, or NO_2_—inside the studied spaces will also have a positive impact on CO_2_ levels. That is, improving air quality in the interior through the elimination of emission spotlights or the implementation of specific ventilation measures to limit and control emission sources will, in turn, imply improving the risk of contagion against COVID. However, when the objective is just to reduce the indoor CO_2_ levels through ventilation, the quality of the ambient air inside and outside the space must also be considered before deciding on the ventilation measures. Reducing ventilation can favour the indoor accumulation of contaminants when there exist internal sources and, although a high rate of air exchange with the outside guarantees that virus transmission is minimized, can also compromise the acceptable indoor levels of contamination if there are external emission sources of pollutants. Therefore, the proposed improvement strategies or alternatives must reach a compromise between the health risk due to COVID-19 and the risk due to exposure to contamination.

Table 10 indicates the worrisome situations of the spaces; areas with the presence of a problem are marked with an X. The detailed recommendation analysis for each of the studied spaces is, however, out of the scope of this paper.

## 4. Conclusions

The COVID-19 pandemic has encouraged authorities and experts to look for useful control strategies to protect citizens and prevent future bio-attacks. Two of the recognized tools to help improve the health of residents and protect them from the risk of spreading infection in places of entertainment, education, residence, and transportation are adequate ventilation and good indoor air quality.

This work develops a novel methodology to continuously evaluate the efficiency of ventilation and the quality of indoor and outdoor air in different scenarios using modern rapid response sensors (RRS). Seven spaces of different types and characteristics, representative of typical public concurrence spaces, were used as study cases to check the validity and usefulness of the different tools of this methodology.

The new methodology includes, in the first place, a quantitative method, based on the dilution principle, to estimate the real air changes per hour (ACH) in the space from simple CO_2_ experimental RRS measurements, the volume of the space, its occupation, and the activity inside it. The box mass balance model which drives the calculation is well-known, but it is used here in an innovative way. One of the main advantages of this methodology is the use of RRS devices for obtaining measurements from which to compute ventilation and indoor air quality rates. These affordable and easy-to-use devices can be used continuously, if desired. Also, the procedure improves on the existing standards, which imply the release of tracer gases forcing to the air exchange to be tested in empty spaces under stationary state conditions. The new methodology does not require very specific conditions (such as closing the premises to public) or the intervention of specialists. The computations can be easily carried out and are compared dynamically with the ACH recommendations of several organizations to prevent COVID contagion and provide minimal ventilation. Because these computations depend heavily on the actual occupancy, the methodology also helps with computing the maximum occupancy that will still respect the given recommendations with the current ventilation strategy for the space. Managers can use this information to decide on the adequacy of the space’s strategy or to limit its occupancy. Furthermore, additional tables are provided to show the occupancy that is allowed should the space implement the ACH recommended by standard design guidelines. Comparison of the data shows that the use of the calculations proposed by this work, based on dynamic real-time measurements from RRS, can help improve the ventilation strategy to increase health protection in a more energy-efficient way. The proposed methodology is simple to apply, allowing non-specialists to be trained for it. A control system that continuously measures and automatically adapts the general ventilation to changes in indoor occupancy and activity could also be implemented. A freely accessible Web page has been created to facilitate the computations involved in this part of the methodology.

Air quality has also been analysed by comparing experimental concentrations of atmospheric pollutants measured inside and outside of the case-study spaces with RRS. The indoor measurements obtained were checked to verify whether the recommended exposure limit values established by the different directives were met. In the situations in which these limits were exceeded, the I/O ratio was computed to help identify the main indoor emission sources and/or sinks. In the presence of indoor sources, structural or activity changes should be recommended. Whenever a problem originates where there is a high outdoor pollution level, depuration measures should be prescribed, and mitigation measures to reduce outdoor environmental pollution should also be addressed by local authorities.

Finding the right strategy for a given space requires a balance among the ventilation needed to reduce the risks of transmission of diseases by viruses or bacteria, the reduction in the potential infiltration of contamination from outside air, and the energy efficiency needed to maintain inside comfort. In line with the results of this paper, after evaluating the ventilation conditions and air quality of all spaces, each space must be considered individually to make a more precise diagnostic of the efficiency of its ventilation system, as well as of the possible causes for high values of pollutants. Combining both perspectives, with separate measures to control the risk of contagion and to ensure good air quality, incompatibilities may appear and will need to be considered. This sets the basis for a future work in which the primary objectives would be to find an equilibrium between ventilation measures that reduce the risk of COVID-19 contagion and ensuring good air quality in the spaces studied. The methodology introduced in this paper is, thus, a valuable tool to help managers of public concurrence spaces to achieve this balance.

## Figures and Tables

**Table 1:** Selected recommendations of air changes per hour (ACH) for public spaces [18].

SPACE	ACH	SPACE	ACH	SPACE	ACH	SPACE	ACH
Auditorium	6–8	Library	4–5	Restaurant	8–12	Gyms	4–6
Lecture room	5–7	Office	4–8	Cinema room	5–8		

**Table 2:** Average exhalation rates of a person, as a function of age, gender, and activity [52].

Activity		Rest		Walking		Physical Activity	
	Gender	Men	Women	Men	Women	Men	Women
Age Groups							
11–15 years		0.0041 l s^−1^	0.0035 l s^−1^	0.0068 l s^−1^	0.0058 l s^−1^	0.0136 l s^−1^	0.0117 l s^−1^
16–20 years		0.0045 l s^−1^	0.0036 l s^−1^	0.0075 l s^−1^	0.0059 l s^−1^	0.0150 l s^−1^	0.0119 l s^−1^
21–29 years		0.0048 l s^−1^	0.0038 l s^−1^	0.0080 l s^−1^	0.0063 l s^−1^	0.0160 l s^−1^	0.0126 l s^−1^
30–39 years		0.0046 l s^−1^	0.0035 l s^−1^	0.0076 l s^−1^	0.0059 l s^−1^	0.0152 l s^−1^	0.0118 l s^−1^
40–49 years		0.0046 l s^−1^	0.0036 l s^−1^	0.0077 l s^−1^	0.0060 l s^−1^	0.0155 l s^−1^	0.0119 l s^−1^
Between 11 and 49 years		0.0042 l s^−1^	0.0036 l s^−1^	0.00752 l s^−1^	0.00598 l s^−1^	0.01506 l s^−1^	0.01198 l s^−1^
Average person		0.00390 l s^−1^		0.00675 l s^−1^		0.01352 l s^−1^	

**Table 4:** Reference environmental limit values.

	PM_2.5_ (µg m^−3^)	PM_10_ (µg m^−3^)	VOCs (ppb *v*/*v*)	NO_2_ (ppb *v*/*v*)
Outdoor	20 ^a^	50 ^a^	80 ^d^	106 (200 µg/m^3^) ^a^
Indoor	15 ^c^	50 ^c^	142 ^c^ (500 µg/m^3^)	74 (140 µg/m^3^) ^b^

Royal Decree 102/2011, ^a^ ANNEX I and ^b^ ANNEX II [57]. ^c^ International Well Building Institute, VOC expressed as N-hexane (86 g/mol) [58]. ^d^ Recommendations according to levels collected by Flow 2.0 device [34].

**Table 5:** Experimental data and computation of $ACH_{real}$ and the recommended $\left[ACH_{ac}\right]$ and $\left[ACH_{mv}\right]$ for each of the spaces with the given occupancy.

SPACE	Experimental Data								Avoid COVID-19 Contagion (ac)		Ensure Minimum Ventilation (mv)			Type of Ventilation
	V $\left(m^{3}\right)$	n	$Exh_{rate}$ $\left({l\;s}^{-1}\right)$	$G_{CO_{2}}$ $\left(m^{3\;}h^{-1}\right)$	$C_{ss}$ (ppm)	$C_{o}$ (ppm)	$ACH_{real}$ $\left(h^{-1}\right)$	Fb	$ACH_{ac}$ $\left(h^{-1}\right)$	$\left[ACH_{ac}\right]$ $\left(h^{-1}\right)$	Fb	$ACH_{mv}$ $\left(h^{-1}\right)$	$\left[ACH_{mv}\right]$ $\left(h^{-1}\right)$	
City study room	77.4	2	0.0039 ^1^	0.02808	628	447	2.004	14	1.30	2	5	0.47	1	Natural ventilation (main door open, windows closed)
University study room	742.2	18	0.0039 ^1^	0.25272	529	489	8.513	14	1.22	2	5	0.44	1	Natural ventilation (doors open, windows 25% open)
Coffee shop	255.5	9	0.00447 ^2^	0.144828	558	507	11.115	15	1.90	2	5	0.63	1	Natural ventilation (door open, roof 50% open)
Restaurant	509.2	17	0.00447 ^2^	0.273564	624	374	2.149	15	1.80	2	5	0.60	1	Natural ventilation (doors open, windows 50% open)
Cinema room	2155.9	20	0.0039 ^1^	0.2808	697	474	0.584	10	0.33	1	5	0.17	1	Natural (main door open, no windows) and forced (Off) ventilation
Gym	2543.1	6	0.01352 ^3^	0.292032	502	475	4.253	15	0.13	1	5	0.04	1	Natural (doors and windows open) and forced (Off) ventilation
Office	64.9	2	0.0039 ^1^	0.02808	548	545	144.222	14	1.55	2	5	0.55	1	Purifier and forced ventilation (On)

Note: $Exh_{rate}$ for people at rest ^1^, rest (80%) and walking (20%) ^2^, or engaging in physical activity ^3^.

**Table 6:** Maximum occupancy for the measured $ACH_{real}$ of each space and compliance with the different guidelines.

		Maximum Occupancy to Avoid COVID-19 Contagion (ac)						Maximum Occupancy to Ensure Minimum Ventilation (mv)					
		$ACH_{real}\geq \left[ACH_{ac}\right]$			$C_{ss}\leq 700$ ppm			$ACH_{real}\geq \left[ACH_{mv}\right]$			$C_{ss}\leq 1000$ ppm		
SPACE	$ACH_{min}$	*n*	$G_{CO_{2}}$ $\left(m^{3\;}h^{-1}\right)$	$C_{ss}$ (ppm)	*n*	$G_{CO_{2}}$ $\left(m^{3\;}h^{-1}\right)$	$C_{ss}$ (ppm)	*n*	$G_{CO_{2}}$ $\left(m^{3\;}h^{-1}\right)$	$C_{ss}$ (ppm)	*n*	$G_{CO_{2}}$ $\left(m^{3\;}h^{-1}\right)$	$C_{ss}$ (ppm)
City study room	2.004	3	0.04	718.50	**2**	0.03	628.00	8	0.11	1171.00	**6**	0.08	990.00
University study room	8.513	117	1.64	749.00	**94**	1.32	697.89	329	4.62	1220.11	**229**	3.22	997.89
Coffee shop	11.115	52	0.84	801.67	**34**	0.55	699.67	156	2.51	1391.00	**87**	1.40	1000.00
Restaurant	2.149	**18**	0.29	638.71	22	0.35	697.53	56	0.90	1197.53	**42**	0.68	991.65
Cinema room	0.584	**0**	0.00	474.00	20	0.28	697.00	**0**	0.00	474.00	47	0.66	998.05
Gym	4.253	188	9.15	1321.00	**50**	2.43	700.00	565	27.50	3017.50	**116**	5.65	997.00
Office	144.222	185	2.60	822.50	**103**	1.45	699.50	519	7.29	1323.50	**303**	4.25	999.50

**Table 7:** Maximum occupancy for the recommended design minimum and maximum ACH of each space and compliance with the different COVID guidelines.

		Maximum Occupancy to Avoid COVID-19 Contagion (ac)							Maximum Occupancy to Avoid COVID-19 Contagion (ac)					
		$ACH_{min}\geq \left[ACH_{ac}\right]$			$C_{ss}\leq 700$ ppm				$ACH_{max}\geq \left[ACH_{ac}\right]$			$C_{ss}\leq 700$ ppm		
SPACE	$ACH_{min}$	*n*	$G_{CO_{2}}$ $(m^{3\;}h^{-1}$)	$C_{ss}$ (ppm)	*n*	$G_{CO_{2}}$ $(m^{3\;}h^{-1}$)	$C_{ss}$ (ppm)	$ACH_{max}$	*n*	$G_{CO_{2}}$ $(m^{3\;}h^{-1}$)	$C_{ss}$ (ppm)	*n*	$G_{CO_{2}}$ $(m^{3\;}h^{-1}$)	$C_{ss}$ (ppm)
City study room	4	6	0.08	719.09	**5**	0.07	673.74	5	7	0.10	700.95	**6**	0.08	664.67
University study room	4	58	0.81	763.29	**44**	0.62	697.08	5	73	1.02	765.18	**55**	0.77	697.08
Coffee shop	10	47	0.76	803.02	**30**	0.48	695.95	12	56	0.90	800.92	**36**	0.58	695.95
Restaurant	8	**75**	1.21	670.27	82	1.32	697.93	12	**113**	1.82	671.59	123	1.98	697.93
Cinema room	5	299	4.20	863.44	**173**	2.43	699.33	8	479	6.73	863.93	**277**	3.89	699.49
Gym	4	188	9.15	1374.53	**47**	2.29	699.88	6	282	13.73	1374.53	**70**	3.41	698.29
Office	4	5	0.07	815.42	**2**	0.03	653.17	8	10	0.14	815.42	**5**	0.07	680.21

**Table 8:** Maximum occupancy for the recommended design minimum and maximum ACH of each space and compliance with the different minimum ventilation guidelines.

		Maximum Occupancy to Ensure Minimum Ventilation (mv)							Maximum Occupancy to Ensure Minimum Ventilation (mv)					
		$ACH_{min}\geq \left[ACH_{mv}\right]$			$C_{ss}\leq 1000$ ppm				$ACH_{max}\geq \left[ACH_{mv}\right]$			$C_{ss}\leq 1000$ ppm		
SPACE	$ACH_{min}$	*n*	$G_{CO_{2}}$ $(m^{3\;}h^{-1}$)	$C_{ss}$ (ppm)	*n*	$G_{CO_{2}}$ $(m^{3\;}h^{-1}$)	$C_{ss}$ (ppm)	$ACH_{max}$	*n*	$G_{CO_{2}}$ $(m^{3\;}h^{-1}$)	$C_{ss}$ (ppm)	*n*	$G_{CO_{2}}$ $(m^{3\;}h^{-1}$)	$C_{ss}$
City study room	4	17	0.24	1217.93	12	0.17	991.19	5	21	0.29	1208.86	15	0.21	991.19
University study room	4	164	2.30	1264.59	108	1.52	999.75	5	206	2.89	1268.37	135	1.90	999.75
Coffee shop	10	141	2.27	1395.05	78	1.26	998.26	12	170	2.74	1399.25	93	1.50	995.11
Restaurant	8	226	3.64	1266.77	158	2.54	998.15	12	339	5.46	1266.77	237	3.81	998.15
Cinema room	5	598	8.40	1252.88	403	5.66	998.90	8	958	13.45	1253.86	646	9.07	999.87
Gym	4	565	27.50	3178.36	109	5.31	996.53	6	847	41.23	3176.77	164	7.98	998.13
Office	4	14	0.20	1302.16	8	0.11	977.67	8	28	0.39	1302.16	16	0.22	977.67

**Table 9:** Experimental indoor and outdoor mean values of PM_2.5_ (µg m^−3^), PM_10_ (µg m^−3^), VOCs (ppb *v/v*), and NO_2_ (ppb *v/v*) in all studied spaces. Description of the location and the possible emission sources for each site.

SPACE	PM_2.5_ (µg m^−3^)			PM_10_ (µg m^−3^)			VOCs (ppb *v/v*)			NO_2_ (ppb *v/v*)			Location	Outdoor Pollution Source(s)	Indoor Pollution Source(s)
	I	O	I/O	I	O	I/O	I	O	I/O	I	O	I/O			
City study room	2.04	2.12	0.96	6.71	14.75	0.45	519.7	183.3	2.84	20.6	25.6	0.80	In the city centre next to a main avenue and road	Traffic and street	VOCs
University study room	2	2.02	0.990	8.25	8.76	0.94	81.33	50.75	1.60	12.56	26.13	0.48	Inside the university campus, which is located next to a dual motorway	Traffic (intercity road)	VOCs
Coffee shop	3.65	2	1.82	9.52	8.6	1.11	106.11	93.78	1.13	46.78	14.33	3.26	In the city centre next to a main avenue	Traffic and street, tobacco smoke	PM, VOCs, NO_2_
Restaurant	2	2.11	0.95	4.84	7.74	0.63	96.21	68	1.41	42.71	32.67	1.31	In the city centre	Street, tobacco smoke	PM, VOCs, NO_2_
Cinema room	2.19	2.86	0.77	5.37	29.07	0.18	839.55	174.18	4.82	14.73	37.18	0.40	Far from the city centre, next to a parking lot and two restaurants	Traffic (main ring road), restaurants, and parking	VOCs
Gym	2	2	1.00	5.69	5.1	1.12	311.11	170	1.83	7.44	15	0.50	In an urban polygon, next to two car repair services	Traffic	PM, VOCs
Office	2.62	20.17	0.13	19.46	55.16	0.35	118.5	78.38	1.51	274.5	82.75	3.32	Inside a car repair service	Repair works	-

I: indoor level, O: outdoor level, I/O: ratio of indoor-to-outdoor levels.

**Table 10:** Summary of the aspects to improve—ventilation and indoor and outdoor levels of pollutants—in each space. The X marks signal situations that need to be addressed.

	Ventilation		PM_2.5_		PM_10_		VOCs		NO_2_	
SPACE	Low Occupancy	Normal Occupancy	In	Out	In	Out	In	Out	In	Out
City study room		X					X	X		
University study room										
Coffee shop								X		
Restaurant	X	X								
Cinema room	X	X					X	X		
Gym		X					X	X		
Office				X		X			X	X

## Data Availability

The original contributions presented in the study are included in the article, further inquiries can be directed to the corresponding author. The validation data of the sensors presented in this study are openly available in FigShare at https://doi.org/10.6084/m9.figshare.25272541.
